# Enhanced generalized normal distribution optimizer with Gaussian distribution repair method and cauchy reverse learning for features selection

**DOI:** 10.1038/s41598-026-35804-y

**Published:** 2026-02-02

**Authors:** Mohamed Ghetas, Mohamed Abd Elaziz, Mohamed Issa

**Affiliations:** 1 Faculty of Computer Science and Engineering, Galala University, Suez, Egypt; 2 Faculty of Science, Zagazig University, Zagazig, Egypt; 3 Computer and Systems Department, Faculty of Engineering, Zagazig University, Zagazig, Egypt; 4 Faculty of Computer Science and Information Technology, Egypt-Japan University for Science and Technology, Alexandria, Egypt

**Keywords:** Features selection, Generalized normal distribution optimization, Meta-heuristics, Classification, Engineering, Mathematics and computing

## Abstract

The presence of noisy, redundant, and irrelevant features in high-dimensional datasets significantly degrades the performance of classification models. Feature selection is a critical pre-processing step to mitigate this issue by identifying an optimal feature subset. While the Generalized Normal Distribution Optimization (GNDO) algorithm has shown promise in various domains, its efficacy for feature selection is hampered by premature convergence and an imbalance between exploration and exploitation. This paper proposes a Binary Adaptive GNDO (BAGNDO) framework to overcome these limitations. BAGNDO integrates three key strategies: an Adaptive Cauchy Reverse Learning (ACRL) mechanism to enhance population diversity, an Elite Pool Strategy to balance the search process, and a Gaussian Distribution-based Worst-solution Repair (GDWR) method to improve exploitation. The performance of BAGNDO was rigorously evaluated against nine state-of-the-art metaheuristic algorithms on 18 UCI benchmark datasets. The results demonstrate the superior efficacy of BAGNDO, which achieved the highest classification accuracy with the most compact feature subsets in 14 out of 18 datasets. Statistical analysis, including the Wilcoxon signed-rank and Friedman tests, confirmed that BAGNDO’s performance is significantly better, establishing it as a robust and efficient solution for wrapper-based feature selection.

## Introduction

Over the past decade, there has been a significant rise in data sources, including fields such as medicine, business, sports, and industry^1^. Large datasets with redundant features slow down analysis and make model training more computationally expensive. Techniques like feature selection, dimensionality reduction (e.g., PCA), and parallel processing can mitigate these issues.

The curse of dimensionality complicates analysis in high-dimensional data. Feature selection and extraction are used to reduce dimensions, but this becomes complex in machine learning due to vast search spaces and limited training data^2^. The feature selection aims to improve the classifier’s performance while reducing the dataset’ size used to train the model^3^. This technique is widely used in data mining^4^, text classification^5,6^, data classification^7,8^, and image processing^9–11^.

The feature selection process involves two stages: exploration and evaluation. The exploration stage identifies potential feature sets, while the evaluation stage assesses the suitability of the relevant subset of possible features.

Feature selection effectively reduces data dimensionality. Its two main techniques are wrapper methods, which use a model to evaluate features, and filter methods, which select features based on statistical measures^12^. Filter feature selection methods generate feature subsets and evaluate them using a statistical measure, independent of any specific classification algorithm^13^, for example, information gain (IG)^14^, Chi-Square^15^, gain ratio^16^, Relieff, and hybrid Relieff^17–20^. Filter methods are fast and efficient because they use statistical measures to evaluate features without training a machine learning model. Filter methods are fast, scalable, and model agnostic. They use statistical measures to select features without training a model, making them an efficient pre-processing step. This independence also makes them simple to implement, general-purpose for any downstream model, and less prone to overfitting.

The main weakness of filter methods is their independence from the final learning algorithm. Because they select features using general statistical measures, they can miss features that are specifically important for a given model. They also tend to overlook complex interactions between multiple features. This often results in a feature subset that is computationally efficient but leads to lower predictive accuracy compared to methods that directly optimize for the model’s performance.

In contrast, the wrapper technique uses a classification method to measure the accuracy of the relevant feature subset during the search. Wrapper methods typically use K-Nearest Neighbors (KNN)^21^ and Support Vector Machines (SVM)^22^ to evaluate the quality of the obtained feature subsets. The primary advantage of the wrapper method is its superior predictive performance and model-specific optimization.

Unlike filter methods, the wrapper method evaluates feature subsets by directly using the target model’s performance. This ensures the selected features are optimally tailored to that specific algorithm, allowing them to capture complex feature interactions and ultimately achieve higher predictive accuracy. Wrapper methods require optimization algorithms to efficiently navigate the vast number of possible feature subsets, which grow exponentially with features. These guided search strategies, like metaheuristics, systematically explore the space to find high-performing combinations without the infeasible cost of a brute-force search. This approach ensures the selected features are directly optimized for the final model’s performance, not a general statistical score. By evaluating entire subsets at once, it naturally captures complex feature interactions, selecting groups that work best together for the specific algorithm.

There are four primary taxonomies of optimization methods for feature selection, each with distinct strategies for navigating the solution space. Deterministic methods, such as exhaustive search^23^ and branch and bound^24^, operate on fixed rules to guarantee finding the optimal feature subset. It offers the distinct advantage of guaranteed optimality but are severely limited by their prohibitive computational cost, which renders them impractical for all but the smallest feature sets. Sequential search methods, including Sequential Forward Selection and Backward Elimination^25^, offer a more efficient, greedy alternative by iteratively adding or removing features. While it is computationally efficient and simple to implement; however, their greedy, step-by-step nature is a major limitation, as they are prone to becoming trapped in local optima and cannot account for complex interactions due to the nesting effect.

Stochastic local search methods incorporate randomness, through techniques like hill climbing^26^, to escape simplistic local traps. Despite their guided search, these methods are often limited to local optima and produce inconsistent results. Meta-heuristic algorithms were developed to overcome these specific shortcomings. Inspired by natural and physical systems, algorithms like Particle Swarm Optimization^27^ and Genetic Algorithms^28^ balance broad exploration of the search space with focused refinement. This allows them to efficiently find robust, high-performing feature subsets that maximize a model’s predictive accuracy.

Researchers turned to metaheuristics to overcome the flaws of earlier methods. Deterministic approaches became computationally infeasible for large datasets, while simpler stochastic searches were prone to local optima and failed to capture complex feature interactions. Metaheuristics emerged as a necessary solution for a more robust and global search.

Metaheuristics excel at wrapper-based feature selection by balancing broad exploration with focused refinement. Their population-based approach helps them escape local optima and find globally optimal feature subsets. By evaluating entire subsets at once, they effectively capture complex feature interactions, and their flexibility allows them to directly optimize for any model’s accuracy. Metaheuristics provides a powerful framework for finding optimal, minimal feature subsets that maximize model performance, outperforming other techniques. They are widely used in many research fields such as Bioinformatics, Engineering Control^29–31^, Mechanical suspensions system^32^, Cross-Docking systems^33,34^ and Digital Watermarking^35^. For feature selection there were many trials such as shown in the following survey^36^.

The “No Free Lunch” (NFL) theorem^37^ for optimization fundamentally establishes that no single meta-heuristic algorithm can universally outperform all others across every possible problem domain. This principle provides a compelling motivation for the continuous exploration and enhancement of new optimizers, such as the Generalized Normal Distribution Algorithm (GNDO)^38^, for complex tasks like feature selection. GNDO is a new metaheuristic algorithm that models solutions as normally distributed random variables to solve optimization problems.

While the GNDO algorithm is theoretically elegant and simple, it struggles with premature convergence and search imbalance on complex feature selection tasks. Therefore, this research is motivated to specialize and enhance GNDO specifically for wrapper-based feature selection to overcome these limitations. Several optimization problems have been solved using GNDO, such as the voltage source converter (VSC) control of hybrid energy storage systems^39^ and PV Triple-Diode Model Parameter Identification^40^.

The basic GNDO algorithm has two main limitations: it is prone to premature convergence on local optima, and it suffers from an imbalance between its local and global search phases. This often causes a rapid loss of population diversity, hindering its effectiveness for complex problems like feature selection.

To directly address the shortcomings of the basic GNDO, this paper introduces a novel binary adaptive variant named BAGNDO, which incorporates three key enhancement strategies. First, an Adaptive Cauchy Reverse Learning strategy (ACRL) is integrated to combat premature convergence and bolster population diversity. By generating reverse solutions using the long-tailed Cauchy distribution, this strategy forces the algorithm to explore uncharted regions of the search space, while an adaptive elitism mechanism ensures that only the most promising solutions, whether original or reversed, are retained for the next generation. Second, an Elite Pool Strategy is employed to rectify the imbalance between exploration and exploitation. This strategy maintains a dynamic pool containing the three best individuals and a newly constructed “guide individual,” which is a weighted combination of the elites. This provides a richer source of high-quality guidance for the population, preventing over-reliance on a single best solution and promoting a more balanced and effective search.

Finally, the Gaussian Distribution-based Worst-solution Repair method (GDWR) is designed to enhance exploitation by directly improving the weakest members of the population. This method uses a Gaussian distribution model, informed by individuals from the elite pool, to perturb and repair poor solutions, effectively pulling them toward more promising regions in the search space and accelerating the convergence toward a refined, high-quality optimum. Collectively, these enhancements work in synergy to overcome the core limitations of the standard GNDO, resulting in a more robust and efficient optimizer for the feature selection task.

The main contributions of this article are the following:

1. The introduction of a novel binary adaptive variant of the Generalized Normal Distribution Optimizer specifically designed to address the feature selection problem.
2. The integration of an Adaptive Cauchy Reverse Learning strategy to increase population diversity, prevent premature convergence, and significantly improve the global exploration capability of the algorithm.
3. The proposal of a Gaussian Distribution-based Worst-solution Repair method that utilizes information from elite individuals to guide and enhance poor solutions, thereby strengthening local exploitation and accelerating convergence.
4. A comprehensive empirical validation on 18 benchmark datasets demonstrating that BAGNDO consistently outperforms competing algorithms by achieving higher classification accuracy with a smaller subset of selected features.

This manuscript is organized into six cohesive sections. Following this introduction, Sect. Related work offers a comprehensive review of the related work in metaheuristic-based feature selection. Section Generalized normal distribution optimization algorithm (GNDO) elaborates on the foundational framework of the Generalized Normal Distribution Optimization (GNDO) algorithm. The proposed binary adaptive GNDO (BAGNDO) approach, including its novel strategies, is detailed in Sect. Proposed BAGNDO. Section 5 is dedicated to the experimental setup, presentation of results, and a thorough discussion of the findings. Finally, Sect. Conclusion concludes the paper by summarizing the key findings and outlining potential directions for future research.

## Related work

The feature selection problem is being addressed by metaheuristic optimization algorithms that use mathematical formulas to guide the search process and allow for random exploration of the domain space^41^. These algorithms are classified based on different perspectives such as memory and memoryless, natural, and non-natural inspiration-based, and local and population-based^42^. Additionally, some researchers categorize them as human-based, and physics-based^43–45^. Swarm intelligence algorithms are a popular nature-based method for feature selection^46–48^. These algorithms mimic the social foraging behavior of animals throughout their lives^49^. Many researchers have proposed different methods to reduce the dimensionality of the feature dataset and obtain high classification accuracy.

In^50^ the authors evaluated the ACO algorithm by using two datasets, liver cancer and breast cancer, and observed that the algorithm could accurately predict the target label. Mafarja and Mirjalili^51^ created a hybrid feature selection method by combining the Whale Optimization Algorithm (WOA) with Simulated Annealing (SA). They developed two models: one where SA is embedded within WOA to refine solutions during the search, and another where SA is applied after WOA to polish the result. The researchers created four hybrid variants by combining WOA with SA, using two hybridization models (LTH and HRH) and two selection strategies (random and tournament). The results showed that all hybrids significantly outperformed the standard WOA. The most effective approach was WOASAT-2 (the High-Level Relay Hybrid with tournament selection), which achieved the highest accuracy and smallest feature subsets, also outperforming other state-of-the-art algorithms like PSO and GA.

^52^ proposed novel Binary Arithmetic Optimization Algorithms (BAOAs) for feature selection. Since the original AOA is for continuous problems, the authors created binary versions using transfer functions (S-shaped and V-shaped) and enhanced them with Lévy flight to escape local optima. The top-performing algorithm was BAOA_S1LF, which uses an S-shaped transfer function and Lévy flight. It achieved the best balance of high classification accuracy and small feature subsets, despite a slight increase in computational time. Based on rigorous comparison against twelve established algorithms (like BPSO and BGWO) on 26 datasets, BAOA_S1LF was statistically proven to be a superior feature selection method, excelling in solution quality, accuracy, and feature reduction. Future work will aim to reduce its computational cost.

An enhanced version of the Capuchin Search Algorithm (CapSA)^53^ for feature selection, named ECapSA. To overcome the limitations of the basic Capuchin Search Algorithm (CapSA), the authors developed an enhanced version (ECapSA) with four key improvements: chaotic inertia weight, sine cosine coefficients, a stochastic learning strategy, and Lévy flight. When tested on 16 datasets, ECapSA significantly outperformed several other well-known algorithms, achieving higher classification accuracy and better fitness. Its superior performance was statistically confirmed, ranking it first overall.

In^54^, the Monarch Butterfly Optimization (MBO) algorithm was applied to wrapper-based feature selection. Using a KNN classifier for evaluation, MBO was tested on 18 UCI datasets and compared to algorithms like PSO, GA, and WOASAT. The results showed that MBO is highly effective, achieving a strong balance between exploration and exploitation. It matched the high accuracy of other methods (avg. 88%) while significantly reducing the number of selected features and demonstrating fast convergence. It is concluded to be a competitive and efficient optimizer for feature selection.

^2^a binary version of the Horse Herd Optimization Algorithm (BHOA) was developed for feature selection. To adapt the continuous algorithm to this binary problem, the authors created 15 variants using three types of transfer functions (S, V, U-shaped) and three crossover operators. The performance of these BHOA variants was thoroughly tested on 24 real-world datasets using six different evaluation metrics. The variant BHOA_S4_Cr1 (using an S-shape transfer function and one-point crossover) was the most effective, achieving the highest accuracy on many datasets and demonstrating strong robustness. In a broad comparison against 21 other methods, BHOA_S4_Cr1 performed on par with the best existing algorithms, a result confirmed by statistical tests. The study concludes that this binary version of HOA is a highly viable and effective method for feature selection.

The Reptile Search Algorithm is a recently developed method that mimics the social behavior of crocodiles^55^. Researchers used RSA to solve the problem of feature selection and tested its performance using sixteen UCI datasets. The results showed that RSA was able to identify the smallest set of selected features, while maintaining high levels of classification accuracy. The authors in^56^ presented the binary golf wolf optimization (BGWO) algorithm with the K-Nearest Neighbors (KNN) classifier to determine the optimal feature for eight benchmark datasets in the UCI repository. By comparing the performance of BGWO with other algorithms such as PSO and GA, the researchers found that BGWO outperformed these other approaches in terms of number of selected features and obtained accuracy. Thus, many researchers suggest that BGWO address the problem of features selection in machine learning tasks.

In a study conducted by the authors in^57^, they introduced the Slap Swarm Algorithm (SSA) along with the SVM to classify three medical datasets obtained from the UCI database repository. The findings of the experiment demonstrated that SSA outperformed well-known metaheuristic algorithms such as PSO GA in terms of classification accuracy. The findings suggest that SSA has the potential to be an effective method for solving many machine learning problems in the medical field.

The authors in^58^ enhance the Black Window Optimization (BWO) algorithm by incorporating a mate selection strategy that aims to overcome issues such as low accuracy, getting stuck in local optima, and slow convergence. To evaluate the performance of the algorithm, the researchers test it on 12 standard datasets from the UCI repository and compare its results with other methods in the literature. Analysis of the results shows that the algorithm significantly improves classification accuracy while reducing the dimensionality of the original dataset. A new approach to classify biomedical data was proposed using the chimp optimization algorithm (ChoA) for wrapper-based feature selection^59^. The algorithm was presented in two versions. In the first version, V-shaped transfer function is used to convert ChoA from the continuous domain to the binary domain. In the second version, a crossover operator was introduced to enhance the exploration behavior of the algorithm. The results of the experiments showed that the proposed method efficiently eliminates irrelevant features, leading to improved classification accuracy.

The Superb Fairy-wren Optimization Algorithm (SFOA)^60^ models its search strategy on the species’ social behaviors, including young bird development, cooperative feeding, and predator evasion. Empirical tests confirm that the bio-inspired SFOA is a robust and efficient algorithm for high-dimensional feature selection. It successfully balances exploration and exploitation within a wrapper method, demonstrating competitive performance on complex datasets and benchmark problems.

In^61^, A novel hybrid IDS framework was developed for efficient botnet detection. It uses a three-stage pipeline: K-NN for pre-processing, a hybrid SFO-WOA model for feature selection, and PSO-K-means for detection. This SFO-WOA-PSO-K-means method achieved near-perfect accuracy (up to 99.8%) and the fastest execution times on benchmark datasets, outperforming other ML models and proving to be a highly effective solution.

A novel HGNDO-EOBL algorithm^62^, which combines a generalized normal distribution optimizer with elite oppositional learning, was shown to be highly effective. It outperformed other meta-heuristics on eight engineering benchmarks, demonstrating its strength for complex optimization tasks.

GNDO^38^ is a metaheuristic that leverages the theoretical foundation of the Gaussian distribution to guide its search process for solving complex optimization problems. The NFL theorem fundamentally justifies the exploration of novel optimizers like GNDO, as no single algorithm is universally superior, and its demonstrated promise in continuous optimization problems suggests potential for adaptation to complex, high-dimensional domains such as feature selection. However, the core limitations of the standard GNDO architecture present a clear impetus for enhancement. Specifically, its proneness to premature convergence and an inherent imbalance between exploration and exploitation are acutely magnified in the vast, combinatorial search landscapes of feature selection, often resulting in convergence to suboptimal local solutions. While existing variants and hybrids have attempted to address these issues, they have not fully bridged the gap, as they remain largely validated on mathematical or specific engineering problems, introduce additional complexity, or apply only partial fixes. Therefore, the primary motivation for enhancing GNDO lies in overcoming these intrinsic weaknesses through more holistic and sophisticated strategies.

Consequently, as follows, a series of subsequent studies have sought to enhance GNDO’s performance by addressing its core limitations. In^63^, The novel hybrid algorithm GNDAOA, which integrates the Arithmetic Optimization Algorithm (AOA), the Generalized Normal Distribution Optimizer (GNDO), and Opposition-based Learning (OBL), was proposed. It outperformed established algorithms on 93% of benchmark functions and showed a performance improvement in data clustering applications.

In^64^, a novel deep learning framework for crop yield prediction integrates a modified optimizer (MSGNDO) with a stacked LSTM-CRF model. MSGNDO simultaneously performs feature selection and model parameter optimization. The framework achieved higher accuracy with lower error than standard models like CNN and LSTM, demonstrating its effectiveness for complex agricultural forecasting.

The elite-driven EDGNDO algorithm enhances the original GNDO with a new search mechanism using elite archives. It is simple, requiring only population size and termination criteria. Tests on benchmarks and engineering problems showed EDGNDO outperforms GNDO and other algorithms in both solution quality and efficiency.

A two hybrid algorithms that merge the GNDO with the Sine Cosine Algorithm (SCA) to improve both exploration and exploitation was proposed^65^. The hybrid GNDO-SCA algorithm, enhanced with an additional weight parameter, demonstrated highly competitive performance on 23 mathematical optimization problems compared to other metaheuristics.

In^66^, A hybrid GNDOA-FCM clustering method was proposed, using a normalized normal distribution to enhance Fuzzy C-Means. It employs the Calinski-Harabasz index to find the optimal cluster count. The method demonstrated higher clustering quality and effectiveness than conventional techniques on benchmark datasets.

A study introduced MOGNDO^67^, which is enhanced with an archive and a leader selection mechanism, was proposed. It significantly outperformed algorithms like MOPSO and MOGWO on benchmarks, producing superior Pareto fronts with excellent convergence, diversity, and coverage. An enhanced feature selection approach called Dynamic Generalized Normal Distribution Optimization (DGNDO)^68^. The authors build upon the recently developed GNDO algorithm, which draws inspiration from normal distribution theory. While GNDO has shown promise in parameter extraction problems, it suffers from limitations when applied to high-dimensional feature selection, including premature convergence to local optima and poor solution diversity.

To overcome GNDO’s limitations, a hybrid algorithm called DGNDO was created by adding a dynamic Local Search Algorithm (LSA). This LSA enhances the best solution, improves random solutions for diversity, or does both, leading to a better balance between exploration and exploitation. Tested on 20 datasets, DGNDO significantly outperformed seven other algorithms (including GNDO, PSO, and GA) by achieving higher accuracy, selecting fewer features, and converging faster.

In^69^, GNDO was successfully applied to optimize the placement and sizing of solar PV sources and D-STATCOMs in power grids. It achieved over 35% power loss reduction on test systems and required less computational effort than other benchmark algorithms.

In^39^ An enhanced GNDO algorithm, NSGNDO, was developed for PV model parameter extraction. By incorporating local and global neighborhood search strategies, it achieved superior accuracy with minimal error and outperformed other metaheuristics, providing a robust solution for precise PV modeling.

GNDO was used to optimize the economic, operational, and environmental performance of Battery Energy Storage Systems (BESS) in AC microgrids under both on-grid and off-grid scenarios^70^. The GNDO-based energy management strategy outperformed CGA, PSO, and JAYA, achieving significant improvements in cost (1.43%), loss (4.44%), and emission (0.18%) reduction, validating its robustness for battery energy storage system management.

Besides the parametric estimation of single-phase transformers as a nonlinear optimization problem, aiming to minimize the error between calculated and measured electrical variables using GNDO to effectively explore the solution space^71^. Numerical tests on transformers of various power ratings demonstrate that the GNDO approach is more effective and robust than other competing optimizers, confirming its potential for solving complex engineering problems with high performance and low computational effort.

### Research gap

A comprehensive analysis of the existing literature reveals a compelling trajectory towards the use of metaheuristic algorithms for wrapper-based feature selection, with numerous studies attesting to the efficacy of nature-inspired optimizers. Despite these advancements, a critical examination uncovers several persistent and interconnected research gaps that this study seeks to address. The foundational impetus for this work is rooted in the NFL theorem which posits that no single optimization algorithm can be universally superior across all problem domains. This theoretical principle justifies the continuous pursuit of novel and enhanced optimizers, particularly for complex, high-dimensional challenges like feature selection, where the limitations of one algorithm create the opportunity for another to excel.

While the GNDO algorithm has demonstrated considerable promise in solving continuous optimization problems its application to the specific demands of feature selection remains relatively nascent and problematic. The core of this issue lies in the inherent architectural limitations of the basic GNDO algorithm, which is prone to premature convergence and a pronounced imbalance between its exploration and exploitation phases. These weaknesses are acutely magnified in the vast, combinatorial search landscape of feature selection, where the algorithm’s tendency to converge rapidly often results in it becoming trapped in local optima, thereby failing to identify the globally optimal or a near-optimal feature subset. This shortfall highlights a clear gap: the standard GNDO is not inherently well-suited for the discrete, high-dimensional nature of feature selection problems without significant modification.

Although the development of GNDO variants marks a step in the right direction, these efforts have not fully bridged the gap for feature selection. For instance, elite-driven approaches like EDGNDO^72^ and multi-objective versions like MOGNDO^67^ have primarily been validated on mathematical benchmark functions and engineering design problems, leaving their performance on large-scale, real-world feature selection datasets uncertain. Similarly, while hybrids such as the GNDO-SCA^65^ aim to balance search dynamics, they often introduce additional parameters, potentially increasing complexity. The recent Dynamic GNDO (DGNDO) explicitly acknowledges GNDO’s limitations for feature selection and incorporates a local search algorithm, confirming the community’s recognition of the problem. However, a gap remains for a more holistic and intrinsic enhancement of GNDO that goes beyond a single-strategy fix.

Furthermore, a nuanced gap exists in the strategic integration of advanced learning and repair mechanisms specifically within the GNDO framework. While concepts like opposition-based learning and elite pools are known in the broader metaheuristic literature, their implementation in GNDO has been limited. The proposed Adaptive Cauchy Reverse Learning (ACRL) strategy represents a significant evolution over basic oppositional learning, as the long-tailed Cauchy distribution provides a more robust mechanism for escaping local optima and exploring distant, promising regions of the search space. More innovatively, the introduction of a Gaussian Distribution-based Worst-solution Repair (GDWR) method addresses a critical oversight in the original algorithm—the neglect of information from the worst-performing individuals. By constructively using knowledge from an elite pool to rehabilitate poor solutions, this mechanism fosters a more balanced and efficient search process, a sophisticated approach not yet seen in prior GNDO variants like HGNDO-EOBL^64^ or GNDAOA^65^.

## Generalized normal distribution optimization algorithm (GNDO)

The GNDO algorithm has a three-stage process for searching for feasible solutions. In the first stage, the algorithm initializes an individual’s position based on the normal distribution, considering the average position of the individual that is farthest from the optimal position. Consequently, the mean position and standard deviations of all individuals are considerably far away from the optimal position. In the second stage, the algorithm updates an individual’s position so that the average position becomes closer to the optimal position, while gradually decreasing the standard deviation. In the third and final stage, the distances between the mean position, the best position, and the standard deviation reach their minimum values. The GNDO algorithm utilizes two learning strategies: local exploitation and global exploration to find the best solution.

### Local exploitation

The initial population is initially created by.

1 $$\:{x}_{i,j}^{t}={l}_{i}+\left({u}_{i}-{l}_{i}\right)\times\:\:\lambda\:\:\:,\:i=\mathrm{1,2},\dots\:\dots\:.N,\:J=\mathrm{1,2},3\dots\:.,D$$

Where N is the population size, D is the dimension of the problem, $\:{l}_{i}$ and $\:{u}_{i}$ are the lower bound and upper bound in the dimension $\:i$, and $\:{\boldsymbol{\lambda\:}}_{}$ is a random number from normal distribution between 0 and 1.

The algorithm aims to improve the solutions within the current search space for all individuals by updating their positions using the generalized mean position and the generalized standard deviation.

This results in a new position for everyone that can be modeled accordingly.

2 $$\:{\boldsymbol{v}}_{\boldsymbol{i}}^{\boldsymbol{t}}={\boldsymbol{\mu\:}}_{\boldsymbol{i}}+{\boldsymbol{\delta\:}}_{\boldsymbol{i}}\:\times\:\:{\boldsymbol{n}}_{\boldsymbol{i}}\:\:\:\:\:\:\:\:\:\:\:\boldsymbol{i}=\mathrm{1,2},\dots\:\boldsymbol{N}$$

The values of $\:{v}_{i}^{t}$, $\:{\mu\:}_{i}$, $\:{\delta\:}_{i}$, and $\:{n}_{i}$ correspond to the trail vector of individual $\:{x}_{i}^{t}$, the generalized mean position, the generalized standard variance, and the penalty factor. These quantities can be determined through computation using the following formulas:

3 $$\:{\mu\:}_{i}=\frac{1}{3}({x}_{i}^{t}+{x}_{best}^{t}+M)$$

4 $$\:{\boldsymbol{\delta\:}}_{\boldsymbol{i}}=\sqrt{\frac{1}{3}\left[\right({\boldsymbol{x}}_{\boldsymbol{i}}^{\boldsymbol{t}}-\boldsymbol{\mu\:}{)}^{2}+({\boldsymbol{x}}_{\boldsymbol{b}\boldsymbol{e}\boldsymbol{s}\boldsymbol{t}}^{\boldsymbol{t}}-\boldsymbol{\mu\:}{)}^{2}+(\boldsymbol{M}-\boldsymbol{\mu\:}{)}^{2}]}$$

5 $$\:\boldsymbol{n}=\left\{\begin{array}{c}\sqrt{-\boldsymbol{log}\left({\boldsymbol{\lambda\:}}_{1}\right)}\times\:\boldsymbol{cos}\left(2\boldsymbol{\pi\:}{\boldsymbol{\lambda\:}}_{2}\right)\:\:\:\:\:\:\:\:\:\:\:\:\:\:\:\:\:if\:a\le\:b\\\:\sqrt{-\boldsymbol{log}\left({\boldsymbol{\lambda\:}}_{1}\right)}\times\:\boldsymbol{cos}\left(2\boldsymbol{\pi\:}{\boldsymbol{\lambda\:}}_{2}+\boldsymbol{\pi\:}\right)\:\:\:\:\:\:\:\:\:\:\:\:\:\:\:\:\:\:\:\:\:\:otherwise\:\:\:\end{array}\right.$$

6 $$\:\boldsymbol{M}=\:\frac{\sum\:_{\boldsymbol{i}=1}^{\boldsymbol{N}}{\boldsymbol{x}}_{\boldsymbol{i}}^{\boldsymbol{t}}}{\boldsymbol{N}}$$

The mean position and best individual of the current population are represented by M and $\:{x}_{i}^{t}$ best, respectively. Additionally, a, b, $\:{\lambda\:}_{1}$, and $\:{\lambda\:}_{2}$ are two random numbers that fall between 0 and 1.

The performance of GNDO is influenced by three key factors: the generalized mean position µ, the generalized standard deviation δ, and the penalty factor η. The current best individual $\:{x}_{i}^{t}$ best guides other individuals to the global minimum but can still become trapped in local optima. To address this, the mean position M of the current population is introduced into Eq. 3. The generalized standard deviation δ enhances the local search of GNDO, with stronger impact when the distance between the mean position M and the current individual’s position $\:{x}_{i}^{t}$ and best individual $\:{x}_{best}^{t}$ is large. The penalty factor η increases the randomness of δ, thereby improving the search ability of GNDO.

### Global exploration

GNDO explores the entire search space to find all possible solutions. The exploration process involves selecting three individuals randomly from the current population to create new offspring. The creation process can be summarized as follows: first, three individuals are selected from the population and named $\:{x}_{p1}^{t}$, $\:{x}_{p2}^{t}$, and $\:{x}_{p3}^{t}$. Then, two trail vectors $\:{v}_{1}$ and $\:{v}_{2}$ are shown in Eq. (7) and Eq. (8). Next, the trail vector $\:{v}_{i}^{t}$ for the current individual is computed using Eq. (9). Finally, the position of the current individual is updated based on the screening mechanism as per Eq. (10).

7 $$\:{\boldsymbol{v}}_{1}=\left\{\begin{array}{c}{\boldsymbol{x}}_{\boldsymbol{i}}^{\boldsymbol{t}}-{\boldsymbol{x}}_{\boldsymbol{p}1}^{\boldsymbol{t}}\:\:\:\:\:\:\:\:\:if\left({\boldsymbol{x}}_{\boldsymbol{i}}^{\boldsymbol{t}}\right)<f\left({\boldsymbol{x}}_{\boldsymbol{p}1}^{\boldsymbol{t}}\right)\\\:{\boldsymbol{x}}_{\boldsymbol{p}1}^{\boldsymbol{t}}-{\boldsymbol{x}}_{\boldsymbol{i}}^{\boldsymbol{t}}\:\:\:\:\:\:\:\:\:\:\:\:\:\:\:otherwise\:\end{array}\right.$$

8 $$\:{\boldsymbol{v}}_{2}=\left\{\begin{array}{c}{\boldsymbol{x}}_{\boldsymbol{p}2}^{\boldsymbol{t}}-{\boldsymbol{x}}_{\boldsymbol{p}3}^{\boldsymbol{t}}\:\:\:\:\:\:\:\:\:\:\:\:if\:\:f\left({\boldsymbol{x}}_{\boldsymbol{p}2}^{\boldsymbol{t}}\right)<f\left({\boldsymbol{x}}_{\boldsymbol{p}3}^{\boldsymbol{t}}\right)\\\:{\boldsymbol{x}}_{\boldsymbol{p}3}^{\boldsymbol{t}}-{\boldsymbol{x}}_{\boldsymbol{p}2}^{\boldsymbol{t}}\:\:\:\:\:\:\:\:\:\:\:\:\:\:\:\:\:\:\:\:\:\:\:otherwise\:\end{array}\right.$$

9 $$\:{\boldsymbol{v}}_{\boldsymbol{i}}^{\boldsymbol{t}}={\boldsymbol{x}}_{\boldsymbol{i}}^{\boldsymbol{t}}+\boldsymbol{\beta\:}\:\times\:\left(\left|{\boldsymbol{\lambda\:}}_{1}\right|\:\times\:\:{\boldsymbol{v}}_{1}\right)+\left(1-\boldsymbol{\beta\:}\right)\:\times\:(\left|{\boldsymbol{\lambda\:}}_{2}\right|\:\times\:\:{\boldsymbol{v}}_{2})\:$$

10 $$\:{\boldsymbol{x}}_{\boldsymbol{i}}^{\boldsymbol{t}+1}=\left\{\begin{array}{c}{\boldsymbol{v}}_{\boldsymbol{i}}^{\boldsymbol{t}}\:\:\:\:\:\:\:\:\:\:\:\:\:\:\:\:\:\:\:if\:\:f\left({\boldsymbol{v}}_{\boldsymbol{i}}^{\boldsymbol{t}}\right)<f\left(\:{\boldsymbol{x}}_{\boldsymbol{i}}^{\boldsymbol{t}}\right)\\\:{\boldsymbol{x}}_{\boldsymbol{i}}^{\boldsymbol{t}}\:\:\:\:\:\:\:\:\:\:\:\:\:\:\:\:\:\:\:\:\:\:\:\:\:\:\:\:\:\:\:\:\:\:\:\:otherwise\end{array}\right.$$

Equation (9) involves three parameters, λ1, λ2, and β, each of which is a randomly selected number between 0 and 1, drawn from a standard normal distribution. λ1 is an adjustment parameter that determines how information is shared between local and global search. To ensure optimal performance of the GNDO, it is crucial that the β parameter is well-defined, as it significantly affects the system’s overall performance. Algorithm 1 demonstrates the GNDO process.

**Algorithm 1:**
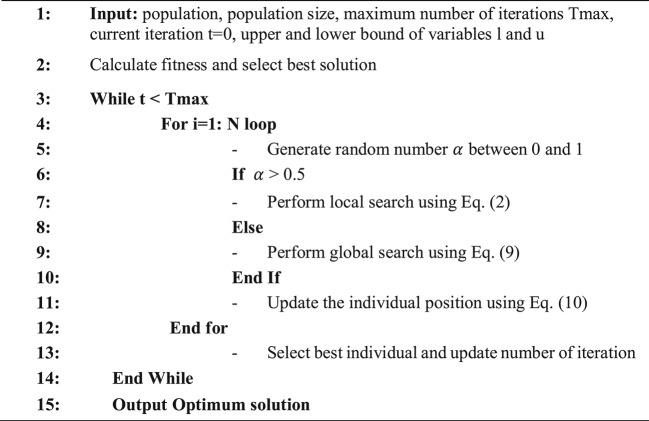
GNDO Algorithm.

## Proposed BAGNDO

The proposed approach aims to address the drawbacks of the conventional GNDO method by introducing three new strategies. Firstly, ACRL approach is implemented to enhance population diversity. Second, to achieve a balance between exploration and exploitation, an elitism pool technique is employed. Finally, GDWR technique is implemented to leverage superior solutions to guide inferior solutions. The following sections demonstrate these strategies in more detail.

### Adaptive cauchy reverse learning strategy (ACRL)

There is a known issue with the GNDO algorithm, which is the loss of diversity in the population as the optimization process continues. To address this problem, several approaches have been proposed by researchers. One commonly used strategy to increase population diversity is the reverse learning approach, which has been extensively studied in literature. This approach utilizes the reverse solution, which has been shown to determine the global optimum to be at least 50% higher than the current solution.

ACRL was chosen according to the following advantages:

1. Enhanced Diversity: ACRL increases the diversity of the population, helping to explore a wider search space and avoid premature convergence.
2. Improved Search Efficiency: By maintaining a diverse population, ACRL enhances the algorithm’s ability to find optimal or near-optimal solutions more efficiently.
3. Robustness: ACRL contributes to the robustness of the optimization process, making the algorithm less likely to get stuck in local optima.
4. Adaptive Mechanism: The adaptive nature of ACRL allows the algorithm to adjust its learning strategy dynamically based on the current state of the population, leading to more effective search processes.
5. Better Convergence: Overall, ACRL helps achieve better convergence rates by ensuring a good balance between exploration and exploitation during the optimization process.

The reverse learning model is well-described and can be used effectively to mitigate the shortcomings of the GNDO algorithm as shown in Eq. (11).

11 $$\:{\boldsymbol{x}}_{\boldsymbol{r}}^{\boldsymbol{t}+1}=\boldsymbol{l}+\boldsymbol{u}-\:{\boldsymbol{x}}_{\boldsymbol{i}}^{\boldsymbol{t}}$$

In fact, improving the diversity of the population depends on the distribution of individuals in the search space. In addition, a more uniform distribution of solutions can lead to better diversity. In optimization algorithms, Cauchy mutation is frequently used to improve convergence. The Cauchy distribution’s fat long tail enables the algorithm to avoid local optimum solutions and prevent premature convergence. The mutation’s value is generated using the Cauchy distribution’s probability density function as described in Eq. (12).

12 $$\:\boldsymbol{f}\left(\boldsymbol{x},{\boldsymbol{x}}_{0},\boldsymbol{\gamma\:}\right)=\:\frac{1}{\boldsymbol{\pi\:}}\:\times\:\:\frac{\boldsymbol{\gamma\:}}{{(\boldsymbol{x}-{\boldsymbol{x}}_{0})}^{2}+\:{\boldsymbol{\gamma\:}}^{2}}$$

The location parameter is represented by $\:{x}_{0}$, while the scaling parameter is represented by γ. To optimize the search process and increase population diversity in the GNDO algorithm, ACRL was incorporated. The ACRL strategy involves using the probability density function of the Cauchy mutation to generate mutation values and generate the new solution as shown in Eq. (13).

13 $$\:{\boldsymbol{x}}_{\boldsymbol{C}\boldsymbol{R}\boldsymbol{L}}^{\boldsymbol{t}}=\boldsymbol{l}+\boldsymbol{u}-\:{{\boldsymbol{c}}_{\boldsymbol{i}}\boldsymbol{x}}_{\boldsymbol{i}}^{\boldsymbol{t}}$$

The $\:{x}_{CRL}^{t}$ is a new solution that corresponds to the existing solution $\:{x}_{i}^{t}$, and the value $\:{c}_{i}\:$represents the mutation value that arises from the Cauchy distribution, which is generated using Eq. (12). The process concludes with the application of an elitist strategy that determines the best individual based on the principles outlined in Eq. (14).

14 $$\:{\boldsymbol{v}}_{\boldsymbol{i}}^{\boldsymbol{t}}=\:\left\{\begin{array}{c}{\boldsymbol{x}}_{\boldsymbol{i}}^{\boldsymbol{t}}\:\:\:\:\:\:\:\:\:\:\:\:\:\:\:\:\:\:\:\:\:\:\:if\:\:f\left({\boldsymbol{x}}_{\boldsymbol{i}}^{\boldsymbol{t}}\right)<\:f\left({\boldsymbol{x}}_{\boldsymbol{C}\boldsymbol{R}\boldsymbol{L}}^{\boldsymbol{t}}\right)\:\\\:{\boldsymbol{x}}_{\boldsymbol{C}\boldsymbol{R}\boldsymbol{L}}^{\boldsymbol{t}}\:\:\:\:\:\:\:\:\:\:\:\:\:\:\:\:\:\:\:\:\:\:\:\:\:\:\:\:\:\:\:\:\:\:\:otherwise\end{array}\right.$$

The performance of the optimization algorithm depends heavily on the initial solution of the swarm intelligence. To improve the quality of the population, the ACRL strategy is utilized to generate initial solutions. Additionally, the reverse solutions corresponding to the ACRL strategy are produced after every iteration, and a screening mechanism is implemented to retain the superior solutions as shown in Eq. (14).

Algorithm 2 outlines the step-by-step process of ACRL strategy. By utilizing this strategy, the quality of the initial solutions, as well as the overall performance of the optimization algorithm, is enhanced.

**Algorithm 2:**
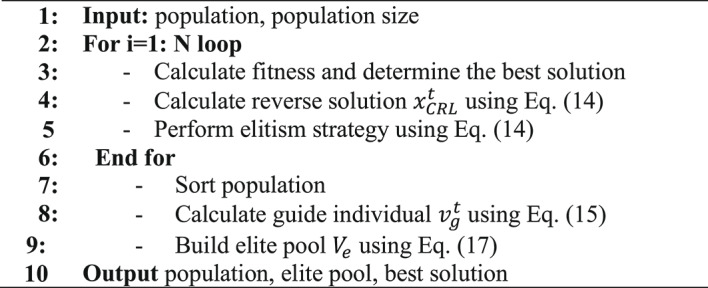
Adaptive Cauchy reverse learning strategy.

### Elite pool strategy

The GNDO technique has a drawback where only the best solution is used to update the positions of other individuals. These speed up convergence but reduce population diversity and increase the risk of getting stuck in a local optimum. The elite pool strategy is introduced to balance exploration and exploitation. This involves placing dominant solutions into the elite pool using Eq. (15). By using this strategy, population diversity is maintained, and the chances of getting trapped in a local optimum are reduced, leading to better overall performance of the optimization algorithm.

15 $$\:{V}_{e}=\{{v}_{best1}^{t},\:{v}_{best2}^{t},\:{v}_{best3}^{t}$$

In the current population, the three best individuals are $\:{v}_{best1}^{t}$, $\:{v}_{best2}^{t}$, and $\:{v}_{best3}^{t}$ who are the most dominant. These individuals are used as a reference to change the position of other individuals in the population. However, relying solely on these dominant individuals can lead to suboptimal solutions, especially if some of the best individuals are stuck in a local optimum. To prevent this, a guide individual is introduced into the elite pool to steer other individuals towards the optimal solution. The guide individual ($\:{v}_{g}^{t}$) is created using the equation in Eq. (16), which improves the convergence efficiency of the optimization algorithm.

16 $$\:{v}_{g}^{t}=\:\alpha\:\:{v}_{best1}^{t}+\:\beta\:\:{v}_{best2}^{t}+\:\delta\:{\:x}_{best3}^{t}$$

Three random numbers, α, β, and δ, are all within the range of [0,1]. The elite pool strategy is consistent and coherent, as it relies on these specific values to generate its results. By using these random numbers, the elite pool strategy can produce accurate and reliable outputs that are useful for creating a variety of $\:{v}_{g}^{t}$. Thus, the elite pool strategy remains consistent and reliable, even when dealing with complex and challenging optimization problems. The final model of the elite pool strategy is.

17 $$\:{V}_{e}=\{{v}_{best1}^{t},\:{v}_{best2}^{t},\:{v}_{best3}^{t},\:{v}_{g}^{t}\}$$

### Gaussian distribution based worst solution repair method (GDWR)

The GNDO method only considers the best solution and disregards information from other dominant populations. However, some researchers believe that we should create distribution models based on the dominant population to exploit their knowledge. This approach involves generating new offspring from a probability model. Unlike other distribution models, this work proposes (GDWR) that fully utilizes the information from elite pool and update the position of individual as shown in Eqs. (18–21)

18 $$\:{x}_{i}^{t+1}=|{v}_{esp}^{t}-\:Gaussian\left(o,\sigma\:\right)|$$

19 $$\:{v}_{esp}^{t}={v}_{e}^{t}\:+r\:\times\:({v}_{e}^{t}-\:{x}_{i}^{t})$$

20 $$\:\sigma\:=\frac{b}{2}\:|{x}_{i}^{t}-{v}_{e}^{t}|$$

21 $$\:b=2-2\times\:\frac{t}{{T}_{max}}$$

Where $\:{v}_{e}^{t}\:$is a solution that is selected randomly from a pool of elite solutions. Meanwhile, the algorithm also makes use of the parameter “b” which gradually decreases to maintain a balance between exploration and exploitation. The value for “t” represents the current iteration, while the maximum number of iterations is denoted by “$\:{T}_{max}$”. Algorithm 3 demonstrates the pseudocode of GDWR method.

**Algorithm 3:**
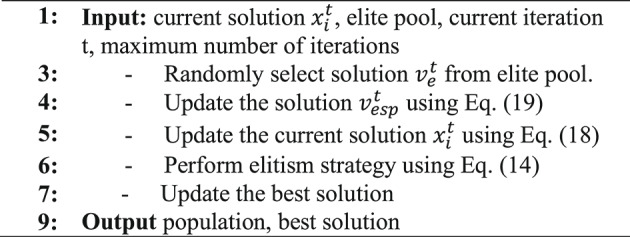
Pseudo-code of GDWR Method.

### BAGNDO for feature selection

Initially, BAGNDO was designed to tackle continuous optimization problems. However, the problem of feature selection requires a binary solution. To solve this problem, BAGNDO converts the continuous solution to binary using transfer functions. The literature suggests different types of transfer methods, including S-shape, V-shape, and U-shape. The S-shape transfer function is the most efficient method for feature selection problem^2^. Therefore, this study employs the S-shape transfer function to obtain the binary version for BAGNDO. The transfer vector(S) can be calculated from the $\:{X}_{i}^{t+1}$ solution using S-shape transfer function as follows.

22 $$\:S\left({X}_{i}^{t+1}\right)=\frac{1}{1+{e}^{-{X}_{i}^{t+1}}}\:\:\:\:\:\:\forall\:i=(\mathrm{1,2},\dots\:\dots\:.D)$$

Where $\:S\left({X}_{i}^{t+1}\right)$ is the probability value of S-shape. The value of each element in the solution $\:{X}_{i}^{t+1}$ is converted either to 0’s or 1’s according to the following equation.

23 $$\:{X}_{i}^{t+1}=\left\{\begin{array}{c}1\:\:\:\:\:\:\:\:rand<S\left({X}_{i}^{t+1}\right)\:\\\:0\:\:\:\:\:\:\:\:\:\:\:\:\:\:\:\:\:\:\:otherwise\end{array}\right.$$

**Fig. 1:**
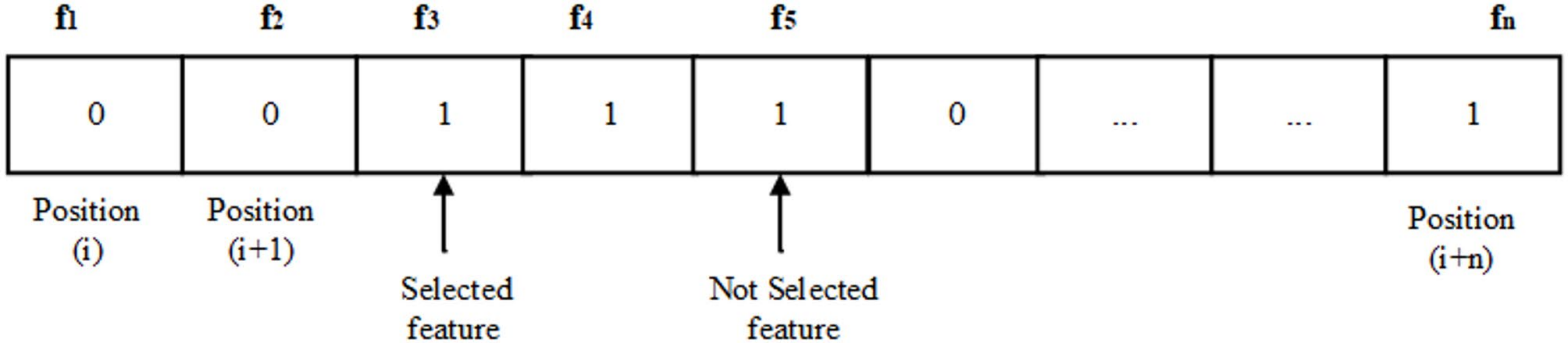
Sample solution where n is the total number of features.

In the binary version, 0 represents an unselected feature, whereas 1 represents a selected feature. Figure 1 illustrates a sample solution with n features.

**Fig. 2:**
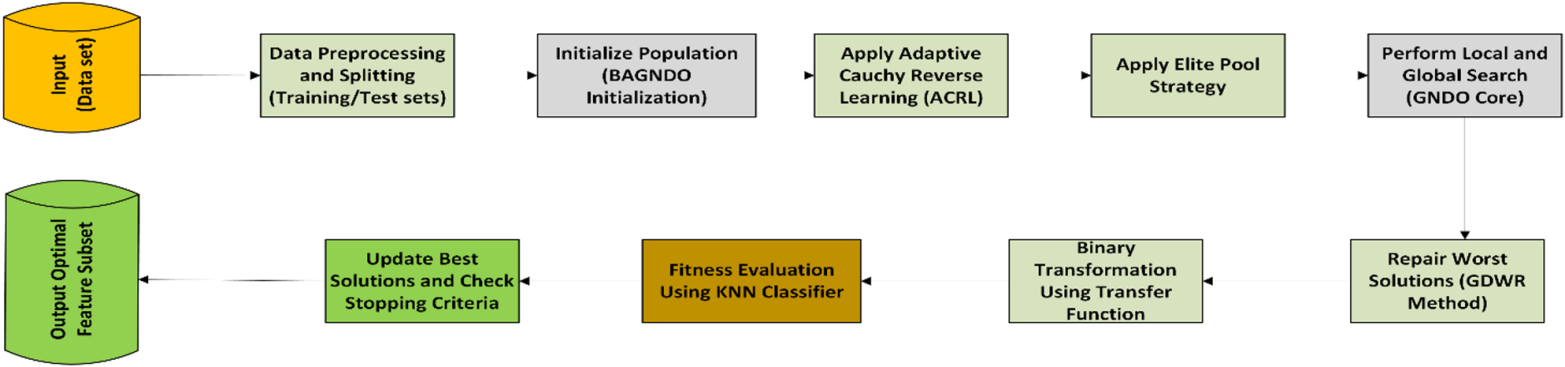
Visualization Flow Diagram of the Proposed BAGNDO Feature Selection Model.

The proposed methods involve splitting the dataset into training and testing sets using cross-validation. The BAGNDO then randomly initializes the population with real numbers and updates the position of everyone using the method demonstrated previously. Once a solution is obtained, the BAGNDO converts it from continuous to discrete space using a transfer function. The solution is then evaluated by the KNN classifier using the testing sets. The BAGNDO receives fitness values from the KNN classifier during each iteration and this process is repeated until the termination criterion is met. On one hand, Fig. 2 show a Visualization flow diagram of the proposed BAGNDO-based feature selection framework showing the interaction among ACRL, GDWR, and the elite pool within the optimization process.

**Fig. 3:**
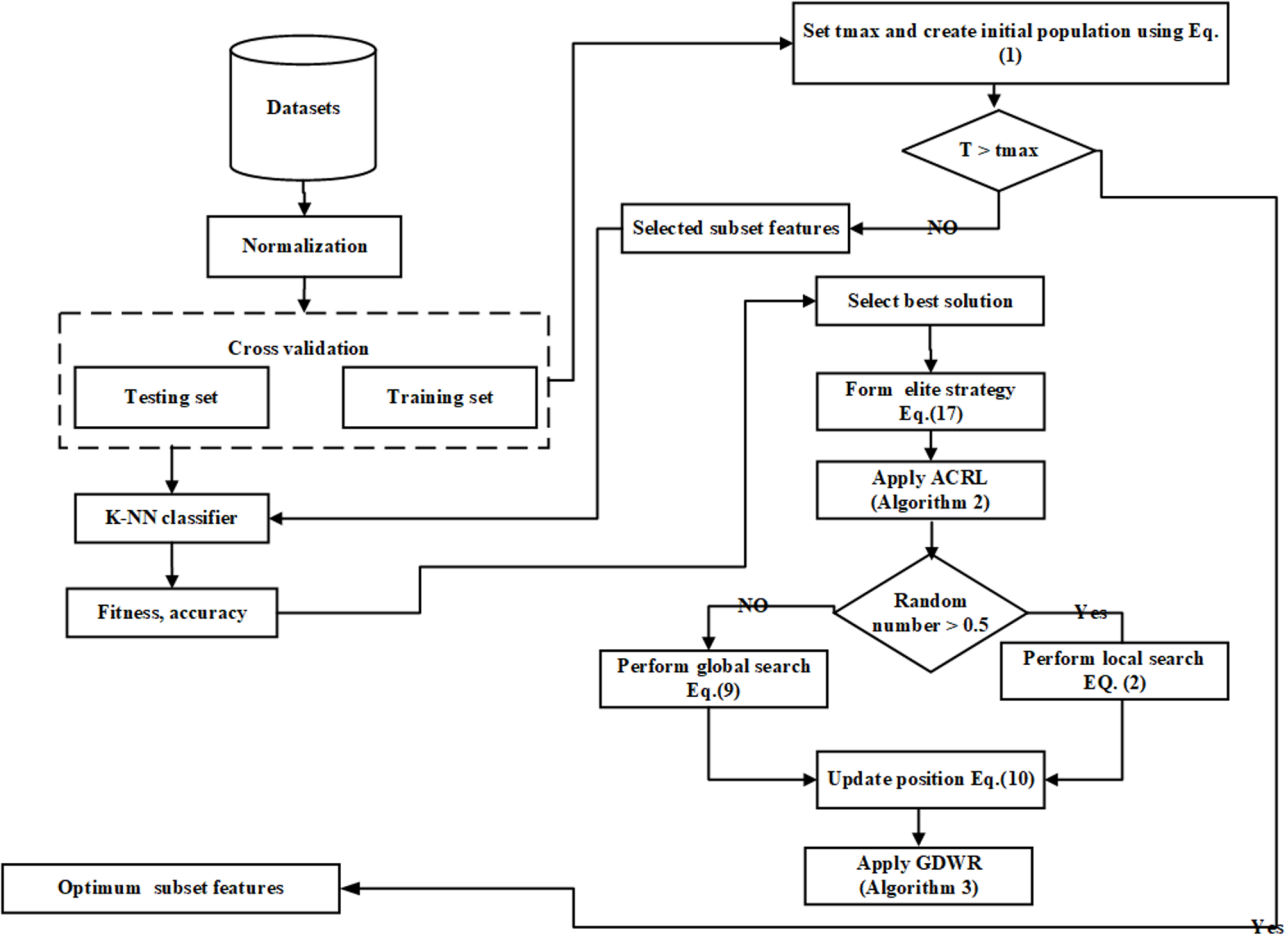
Proposed BAGNDOprocess for feature extraction.

On the other hand, Fig. 3 shows a visual representation of the process, and the pseudo code of the algorithm is depicted in Algorithm 4.

**Algorithm 4:**
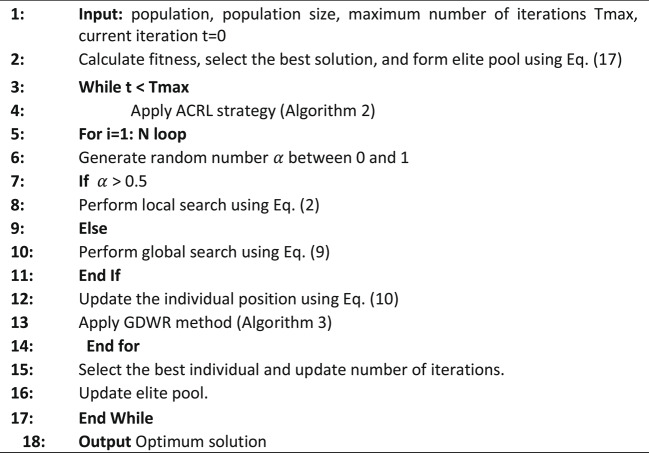
BAGNDO Algorithm.

## Experimental tests and discussion

### Datasets description

MATLAB was used to implement the proposed BAGNDO algorithm, which was then evaluated using 18 benchmark datasets from the UCI data repository. These datasets contain various features and instances from different fields. Table 1 provides details on each dataset, including the number of instances and attributes.

### Parameter setting

The proposed method employs a wrapper feature selection technique that utilizes k-nearest neighbors with k = 5, as described in^2^, to evaluate each solution’s fitness value. Each dataset is split into k-fold cross-validation to achieve this, with the first k-1 folds being used for learning and evaluation and the final fold being utilized for testing. This procedure is carried out M times, resulting in each method being tested on each dataset k*M times. All methods are executed using a set number of iterations (100), a population size of 10, and M set to 5. The experiments were conducted on an Intel Core i7 machine, with 2.2 GHZ and 16GB RAM. All comparative methods were used with the same original parameters.

Table 1 show the parameters setting for different comparative algorithms including ChOA^59^, BGNDO^73^, RSA^55^, SSA, SCA^74^, WOA^75^, GWO^56^, PSO^76^, and AOA^52^.

**Table 1:** Parameters setting for comparative algorithms.

Algorithm	Key Parameters	Typical Values / Notes
ChOA (Chimp Optimization Algorithm)	Population size (N), Max iterations (T), a, b (control coefficients)	*N* = 30–50, T = 100–500, a = 2, b = 1
BGNDO (Binary Generalized Normal Distribution Optimization)	Population size, Max iterations, σ (std. dev.), α (scaling factor)	*N* = 30–50, T = 100–500, σ = 0.1–0.5, α = 0.5–1
RSA (Reptile Search Algorithm)	Population size, Max iterations, step size	*N* = 30, T = 500, step size = 0.1–1
SSA (Salp Swarm Algorithm)	Population size, Max iterations, c1, c2	*N* = 30, T = 500, c1 = 2, c2 = 0.5–1
SCA (Sine Cosine Algorithm)	Population size, Max iterations, r1, r2, r3	*N* = 30, T = 500, r1 = 2, r2 = 2π, r3 = 2
WOA (Whale Optimization Algorithm)	Population size, Max iterations, a (shrinking factor)	*N* = 30–50, T = 500, a = 2 → 0 (linearly decreasing)
GWO (Grey Wolf Optimizer)	Population size, Max iterations, a (convergence factor)	*N* = 30–50, T = 500, a = 2 → 0
PSO (Particle Swarm Optimization)	Population size, Max iterations, w (inertia), c1, c2	*N* = 30–50, T = 500, w = 0.5–1, c1 = c2 = 2
AOA (Arithmetic Optimization Algorithm)	Population size, Max iterations, MOA, MOP	*N* = 30, T = 500, MOA = 0.5–1, MOP = 0.1–0.9

Tables 1 and 2 demonstrates the binary version of PSO, GWO, and WOA in terms of transfer type, threshold, and update rule.

**Table 2:** Binary versions of comparative algorithms.

Algorithm	Transfer Function	Threshold	Update Rule
Binary PSO (BPSO)	Sigmoid: $$\:S\left({v}_{i,j}\right)=\:\raisebox{1ex}{$1$}\!\left/\:\!\raisebox{-1ex}{$(1+{e}^{{-v}_{i,j}})$}\right.$$	Generate random r ∈ [0,1]; if r < $$\:S\left({v}_{i,j}\right)$$→ 1 else 0	$$\:{v}_{i,j}^{t+1}=w.\:{v}_{i,j}^{t}+c1.\:r1\:\left({pbest}_{i,j}-{x}_{i,j}\right)+c2.\:r2.\:({gbest}_{j}-{x}_{i,j})$$
Binary GWO (BGWO)	S-shaped: $$\:\left(x\right)=\:\raisebox{1ex}{$1$}\!\left/\:\!\raisebox{-1ex}{$(1+{e}^{-x})$}\right.$$	tanh(x)	
Binary WOA (BWOA)	Sigmoid or V-shaped transfer functions	r < S(x) → 1 else 0	Position updated using encircling prey and spiral update equations, then binarized using transfer function

### Evaluation criterion

This section discusses the evaluation criteria that can be used to assess the effectiveness of the proposed BAGNDO approach along with other methods. The evaluation metrics that will be used include fitness function, classification error, and average selected feature. These measurements give better understanding of the effeteness of the suggested method and evaluate it against other methods in the literature. The feature selection problem in machine learning involves finding a subset of relevant features to improve model performance. This is a multi-objective problem with contradictory objectives, such as minimizing the number of features while maximizing classification accuracy. To balance these objectives, a function that combines classification error rate (Cer), and ratio between the number of selected features (s) and the total number of features(D) is used. The fitness value is calculated as shown in Eq. (23).

**Table 3:** UCI Benchmarks.

No.	DS Name	Number of instances	Number of features
1	Breastcancer	699	9
2	BreastEW	569	30
3	CongressEW	435	16
4	Exactly	1000	13
5	Exactly2	1000	13
6	HeartEW	270	13
7	IonosphereEW	351	34
8	KrvskpEW	3196	36
9	Lymphography	148	18
10	M-of-n	1000	13
11	PenglungEW	73	325
12	SonarEW	208	60
13	SpectEW	267	22
14	Tic-tac-toe	958	9
15	Vote	300	16
16	WaveformEW	500	40
17	WineEW	178	13
18	Zoo	101	16

23 $$\:Fitness=\:\alpha\:\:Cer+(1-\alpha\:)\:\frac{S}{D}$$

This function is evaluated using KNN classifier and weighted by factors α, which control the balance between feature selection and classification error. Previous work has used a value of α = 0.99 to prioritize lower classification error^2^. The average classification error measure as shown in Eq. (24) is used to calculate the average number of instances that have been classified incorrectly.

24 $$\:{AV}_{Cer}=\:\frac{1}{M}\sum\:_{i=1}^{M}Cer\:,\:Cer=\:\frac{FP+FN}{TP+TN+FP+FN}$$

The terms FP and FN refer to the number of predicted classes that are False Positive and False Negative, respectively. On the other hand, TP and TN refer to the number of classes that are predicted as True Positive and True Negative, and M is the number of independent runs of the algorithm. The average selection size measure computes the average number of selected features as follows:

25 $$\:{AV}_{S}=\:\frac{1}{M}\:\sum\:_{i=1}^{M}s$$

### Results and discussion

This section is divided into subsections, the first one demonstrate a comparative analysis of the performance between the proposed method (BAGNDO), and other feature selection methods, namely, ChOA^59^, BGNDO^73^, RSA^55^, SSA, SCA^74^, WOA^75^, GWO^56^, PSO^76^, and AOA^52^. The second subsection evaluate the individual contribution of each compnent of the proposed method by systematicaly removing it and obsevaing it is effectiveness on the performance.

#### Comparative analysis with state-of-the-art methods

Table 4 displays the meaning of the fitness values, and it is observed that BAGNDO achieved the lowest mean fitness value in all datasets compared to the other methods. This indicates that BAGNDO has the potential to search for the global optimal solution resulting in superior performance. The ACLR learning strategy and GDWR method enabled the algorithm to search for new solutions in the whole space and repair the worst solutions, avoiding local minimum traps. The BAGNDO algorithm outperformed other methods in 14 datasets, while CHOA, GNDO, and PSO outperformed GWSR in 8 datasets.

Table 5 demonstrates that BAGNDO is a robust algorithm that provides consistent results with the smallest standard deviation in 11 out of 18 datasets. BAGNDO methods significantly improved global and local search, contributing to the algorithm’s performance.

**Fig. 4:**
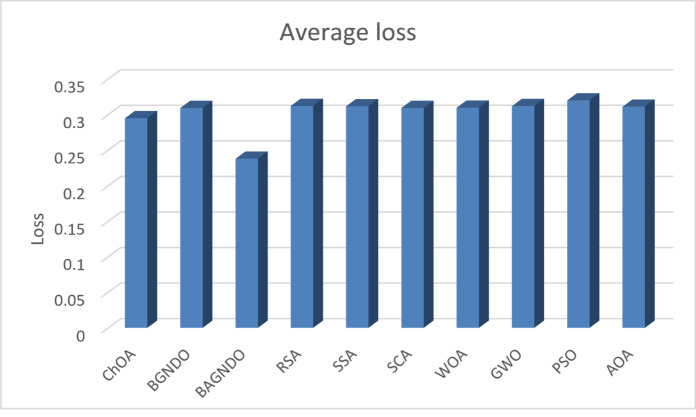
Average loss.

Table 6 displays the classification error results, and BAGNDO algorithm contributed the smallest classification error in 14 datasets, outperforming other methods. The ACRL strategy and GDWR enabled the algorithm to find the best attribute that reduced the classification error. On the other hand, ChOA, and PSO outperformed BAGNDO in 4 datasets, but the difference was not significant. Figure 4 showed that BAGNDO achieved the lowest classification error among the state-of-the-art feature selection algorithm.

**Table 4:** Mean fitness value.

Data Set No	BAGNDO	ChOA	BGNDO	RSA	SCA	WOA	GWO	PSO	AOA	SSA
1	**0.0781**	0.0843	0.0884	0.0905	0.0868	0.0866	0.0901	0.0924	0.0941	0.0920
2	**0.2139**	0.2185	0.2174	0.2232	0.2189	0.2188	0.2219	0.2177	0.2204	0.2190
3	**0.1347**	0.1514	0.1596	0.1643	0.1604	0.1606	0.1607	0.1574	0.1605	0.1634
4	**0.3096**	0.3104	0.3103	0.3104	0.3104	0.3109	0.3109	0.3104	0.3113	0.3113
5	**0.2403**	0.2408	0.2411	0.2411	0.2407	0.2408	0.2414	0.2411	0.2414	0.2414
6	0.2883	**0.2815**	0.2885	0.2929	0.2877	0.2903	0.2900	0.2903	0.2911	0.2938
7	0.2734	0.2793	**0.2710**	0.2795	0.2749	0.2769	0.2778	0.2748	0.2772	0.2790
8	**0.4536**	0.4548	0.4557	0.4560	0.4553	0.4547	0.4563	0.4550	0.4553	0.4556
9	0.2665	0.2683	0.2789	0.2820	0.2813	0.2741	0.2927	**0.2662**	0.2747	0.2867
10	**0.3168**	0.3175	0.3186	0.3241	0.3183	0.3181	0.3186	0.3263	0.3312	0.3187
11	**0.5439**	0.5554	0.5503	0.5591	0.5541	0.5545	0.5579	0.5537	0.5569	0.5544
12	**0.4444**	0.4465	0.4512	0.4518	0.4469	0.4488	0.4578	0.4591	0.4476	0.4650
13	0.2056	0.2058	0.2067	0.2059	0.2064	0.2063	0.2066	**0.2052**	0.2068	0.2068
14	**0.3451**	0.3453	0.3455	0.3455	0.3458	0.3462	0.3460	0.3462	0.3455	0.3460
15	**0.1557**	0.1619	0.1836	0.1906	0.1869	0.1886	0.1901	0.1838	0.1859	0.1871
16	**0.6509**	0.6518	0.6539	0.6541	0.6521	0.6528	0.6523	0.6510	0.6536	0.6543
17	**0.2453**	0.2597	0.2659	0.2686	0.2527	0.2613	0.2649	0.2613	0.2638	0.2605
18	**0.2860**	0.2863	0.2870	0.2866	0.2869	0.2869	0.2873	0.2871	0.2874	0.2870

*Significant values are in [bold].*

**Table 5:** The standard deviation of fitness value.

Data Set No	BAGNDO	ChOA	BGNDO	RSA	SCA	WOA	GWO	PSO	AOA	SSA
1	0.0027	0.0058	0.0060	0.0060	0.0062	0.0065	0.0054	0.0036	**0.0009**	0.0043
2	**0.0014**	0.0036	0.0050	0.0043	0.0037	0.0061	0.0023	0.0054	0.0032	0.0038
3	0.0498	0.0145	**0.0017**	0.0028	0.0023	0.0034	0.0045	0.0032	0.0032	0.0053
4	**0.0000**	0.0005	0.0006	0.0008	**0.0000**	0.0004	0.0004	0.0008	0.0003	0.0003
5	**0.0000**	0.0004	0.0005	0.0000	0.0004	0.0004	0.0004	0.0005	0.0004	0.0004
6	0.0033	0.0078	0.0065	0.0048	0.0065	**0.0020**	0.0068	0.0040	0.0071	0.0024
7	**0.0000**	0.0040	0.0031	0.0063	0.0067	0.0071	0.0035	0.0041	0.0092	0.0053
8	0.0010	0.0017	**0.0008**	0.0011	0.0013	0.0016	0.0019	0.0021	0.0013	0.0011
9	0.0088	0.0185	0.0083	0.0152	0.0082	**0.0062**	0.0077	0.0229	0.0123	0.0113
10	**0.0001**	0.0006	0.0009	0.0116	0.0006	0.0008	0.0013	0.0120	0.0135	0.0003
11	**0.0000**	0.0083	0.0068	0.0089	0.0084	0.0076	0.0101	0.0099	0.0076	0.0110
12	**0.0000**	0.0003	0.0122	0.0142	0.0005	0.0092	0.0203	0.0211	0.0006	0.0228
13	0.0002	**0.0000**	0.0003	0.0006	0.0002	0.0005	0.0004	0.0006	0.0002	0.0006
14	**0.0005**	0.0008	0.0009	0.0009	0.0010	0.0009	0.0006	0.0012	0.0009	0.0010
15	0.0589	0.0545	0.0042	0.0037	**0.0021**	0.0024	0.0039	0.0039	0.0035	0.0062
16	**0.0000**	0.0007	0.0014	0.0015	0.0017	0.0010	0.0017	0.0017	0.0020	0.0017
17	0.0137	0.0126	0.0106	0.0084	0.0197	0.0117	0.0110	0.0178	**0.0073**	0.0232
18	**0.0003**	0.0005	0.0010	0.0005	0.0005	0.0011	0.0005	0.0007	0.0004	0.0007

Significant values are in [bold].

**Table 6:** Average classification error.

Data Set No	BAGNDO	ChOA	BGNDO	RSA	SCA	WOA	GWO	PSO	AOA	SSA
1	**0.0067**	0.0833	0.0876	0.0895	0.0859	0.0858	0.0890	0.0930	0.0930	0.0910
2	**0.1263**	0.2162	0.2144	0.2204	0.2169	0.2158	0.2190	0.2207	0.2172	0.2162
3	**0.0123**	0.1490	0.1563	0.1609	0.1577	0.1582	0.1582	0.1605	0.1577	0.1605
4	**0.2010**	0.3120	0.3120	0.3120	0.3120	0.3120	0.3120	0.3120	0.3120	0.3120
5	0.2420	**0.0241**	0.2420	0.2420	0.2420	0.2420	0.2420	0.2420	0.2420	0.2420
6	0.2852	**0.2800**	0.2859	0.2889	0.2859	0.2881	0.2867	0.2985	0.2889	0.2904
7	0.2707	0.2769	**0.2672**	0.2764	0.2729	0.2741	0.2758	0.2815	0.2746	0.2752
8	**0.2534**	0.4542	0.4551	0.4555	0.4553	0.4539	0.4554	0.4566	0.4546	0.4543
9	**0.2649**	0.2676	0.2770	0.2797	0.2797	0.2730	0.2905	0.2932	0.2730	0.2851
10	**0.2500**	0.3190	0.3190	0.3244	0.3190	0.3190	0.3190	0.3386	0.3314	0.3188
11	**0.3400**	0.5562	0.5498	0.5589	0.5543	0.5543	0.5575	0.5776	0.5566	0.5539
12	**0.4452**	0.4471	0.4503	0.4516	0.4471	0.4487	0.4571	0.4763	0.4471	0.4639
13	**0.1960**	0.2060	0.2060	0.2060	0.2060	0.2060	0.2060	0.2060	0.2060	0.2060
14	**0.2830**	0.3466	0.3466	0.3466	0.3466	0.3466	0.3466	0.3466	0.3466	0.3466
15	**0.1040**	0.1607	0.1813	0.1873	0.1853	0.1867	0.1873	0.1900	0.1833	0.1847
16	0.6530	0.6541	0.6546	0.6566	0.6537	0.6540	**0.6530**	0.6569	0.6557	0.6556
17	**0.2449**	0.2596	0.2652	0.2674	0.2517	0.2607	0.2640	0.2697	0.2629	0.2584
18	**0.0950**	0.2871	0.2871	0.2871	0.2871	0.2871	0.2871	0.3307	0.2871	0.2871

Significant values are in [bold].

**Table 7:** Average number of selected features.

Data Set No	BAGNDO	ChOA	BGNDO	RSA		SCA		WOA		GWO		PSO		AOA		SSA	
1	**1.40**	1.67	1.47	1.67		1.57		1.53		1.80		2.60		1.80		1.70	
2	14.40	13.60	15.40	15.00		**12.60**		15.40		15.40		15.40		16.00		15.00	
3	**4.40**	6.20	7.80	8.00		6.80		6.40		6.60		7.20		7.00		7.20	
4	**1.00**	2.00	1.80	2.00		2.00		2.60		2.60		3.00		3.20		3.20	
5	**1.00**	1.60	2.00	2.00		1.40		1.60		2.40		2.60		2.40		2.40	
6	7.80	**5.60**	7.00	9.00		6.00		6.60		8.00		7.60		6.60		8.20	
7	18.40	17.40	22.00	20.20		**16.00**		19.00		16.20		19.80		18.00		22.40	
8	17.00	18.40	18.60	18.20		**16.60**		19.20		19.60		19.40		19.00		20.80	
9	7.80	**6.20**	8.40	9.20		7.80		7.00		9.20		7.20		8.00		8.00	
10	**2.00**	2.20	3.60	3.80		3.20		3.00		3.60		5.00		4.00		4.00	
11	164.63	**156.63**	196.30	188.70		173.03		185.17		193.87		185.67		189.90		196.07	
12	**22.03**	23.33	32.27	28.43		25.63		27.40		31.93		30.10		29.60		34.10	
13	3.60	4.00	6.00	4.40		5.40		5.20		5.80		**3.40**		6.40		6.20	
14	**1.80**	2.00	2.20	2.20		2.40		2.80		2.60		4.80		2.20		2.60	
15	5.20	**4.60**	6.60	8.20		5.40		6.00		7.40		6.20		7.00		6.80	
16	17.60	17.20	23.20	**16.20**		19.80		21.20		23.40		21.00		17.80		21.00	
17	**3.60**	**3.60**	4.40	5.00		4.60		4.20		4.60		6.00		4.60		6.00	
18	**2.80**	3.20	4.40	3.80		4.20		4.20		4.80		5.60		5.00		4.40	

Significant values are in [bold].

Table 7 presents the average selected subset features, and BAGNDO had the smallest number of selected subset features among the other algorithms in 14 datasets. However, BAGNDO can maintain the minimum classification error in these datasets, which is more valuable in classification problems. The GDWR method enables the algorithm to balance between the number of selected subset features and the classification error, contributing to the superiority of BAGNDO. ChOA can offer fewer subsets of selected features in 4 datasets compared to GWSR.

**Fig. 5:**
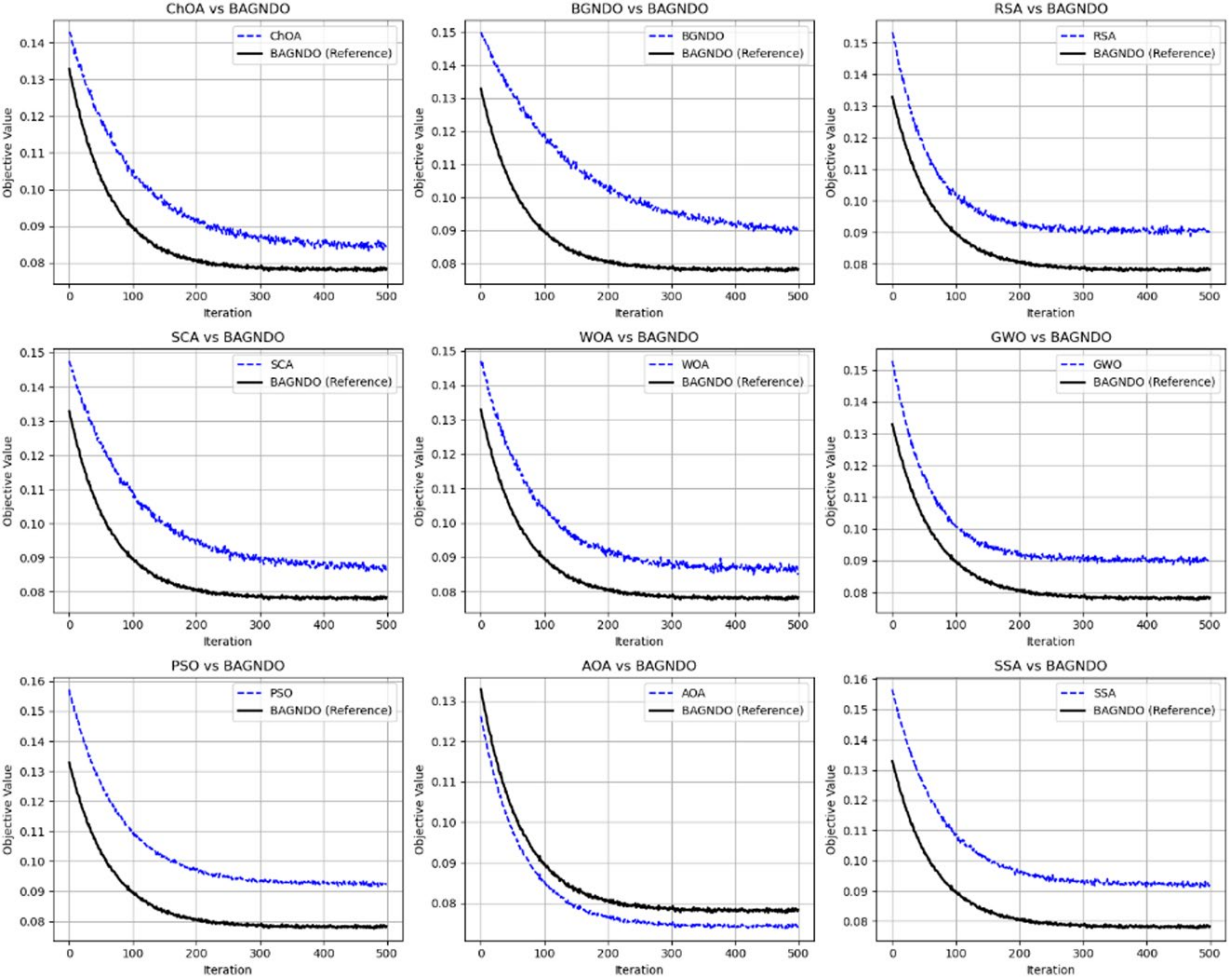
: Convergence curves of BAGNDO compared with another optimization algorithm on breast cancer dataset.

**Fig. 6:**
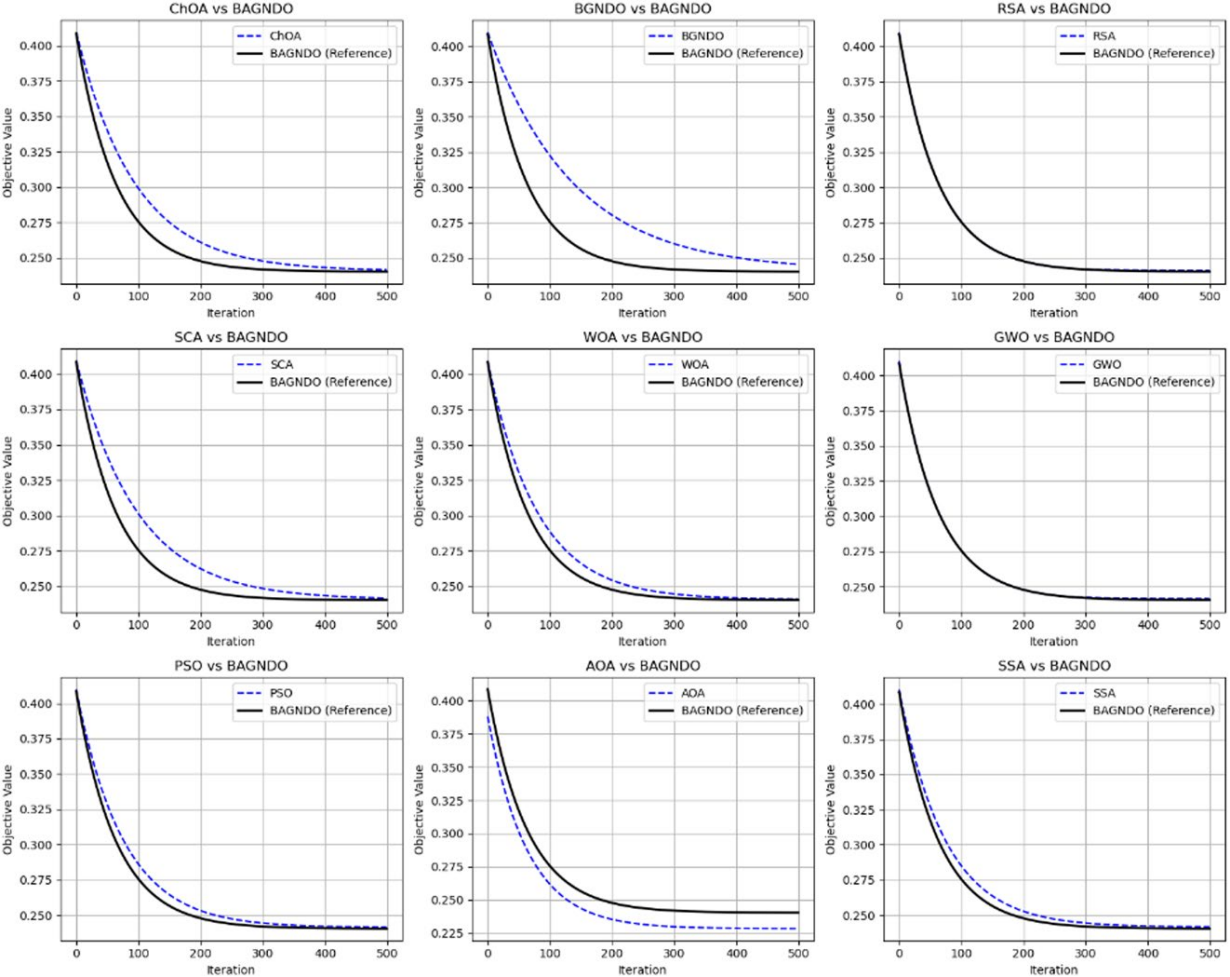
Convergence curves of BAGNDO with another optimization algorithm on Exactly2 dataset.

Figure 5 shows the convergence curve of the proposed BAGNDO algorithm against other optimization algorithms with BAGNDO serving as the reference algorithm for Breast cancer dataset. The figure show that BAGNDO consistently achieves the lowest objective values in 8 out of 9 comparisons, demonstrating its overall superiority in convergence and robustness of BAGNDO relative to other algorithms. Figure 6 illustrates the convergence curves of BAGNDO compared with other algorithms on the Exactly2 dataset highlighting BAGNDO’s superior performance and robustness across different methods.

**Table 8:** The results of Wilcoxon test of BAGNDO with comparative algoithms.

DS No.	ChOA	BGNDO	RSA	SSA	SCA	WOA	GWO	PSO	AOA
1	0.002	0.000	0.000	0.000	0.000	0.001	0.000	0.003	0.003
2	0.008	0.000	0.001	0.000	0.001	0.003	0.003	0.008	0.003
3	0.000	0.000	0.003	0.000	0.004	0.003	0.000	0.000	0.000
4	0.003	0.000	0.003	0.002	0.001	0.000	0.002	0.001	0.004
5	0.002	0.000	0.002	0.000	0.000	0.000	0.000	0.002	0.040
6	**0.060**	0.000	0.007	0.003	0.000	0.000	0.060	0.000	0.002
7	0.001	**0.080**	0.007	0.000	0.004	0.000	0.004	0.003	0.003
8	0.006	0.000	0.000	0.007	0.006	0.000	0.007	0.004	0.070
9	0.000	0.003	0.007	0.004	0.009	0.000	0.002	**0.010**	0.003
10	0.000	0.000	0.002	0.000	0.003	0.002	0.003	0.001	0.000
11	0.004	0.001	0.002	0.000	0.009	0.008	0.004	0.000	0.020
12	0.007	0.003	0.002	0.000	0.000	0.004	0.008	0.000	0.004
13	0.000	0.001	0.003	0.004	0.001	0.006	0.003	**0.040**	0.004
14	0.001	0.004	0.005	0.003	0.001	0.000	0.000	0.004	0.006
15	0.000	0.002	0.050	0.000	0.002	0.003	0.003	0.001	0.004
16	0.005	0.050	0.004	0.001	0.003	0.060	0.001	0.003	0.004
17	0.000	0.000	0.060	0.003	0.000	0.001	0.007	0.030	0.000
18	0.000	0.000	0.003	0.006	0.000	0.002	0.090	0.000	0.001
Win | Loss	17|1	16|2	16|2	18|0	18|0	17|1	16|2	16|2	18|0

Significant values are in [bold].

The performance of two different methods was compared using the Wilcoxon signed-rank test with a confidence level of 0.95%. The results in Table 8 indicate that the methods have similar performance unless the p-value of the test is less than 0.005, which signifies significant differences in performance. Upon careful inspection, the proposed BAGNDO was found to perform significantly better than other methods. For instance, in the 17th dataset, the proposed method outperformed ChOA significantly, while it had the same performance as ChOA in one dataset. Similarly, the proposed method achieved significant performance better than AOA in all datasets. The findings highlight the superiority of the proposed method over the other methods in terms of performance.

**Table 9:** Friedman test results statistical significance among optimization algorithms.

Algorithm	Average Rank	Friedman Statistic	*p*-value
AOA	1	87.12	6.14857E-15
BAGNDO	2	87.12	6.14857E-15
ChOA	3	87.12	6.14857E-15
WOA	4.4	87.12	6.14857E-15
SCA	4.6	87.12	6.14857E-15
GWO	6.6	87.12	6.14857E-15
BGNDO	7.1	87.12	6.14857E-15
RSA	7.3	87.12	6.14857E-15
SSA	9.3	87.12	6.14857E-15
PSO	9.7	87.12	6.14857E-15

Table 9 presents statistical comparisons of the optimization algorithms using Friedman test. The test results were a Friedman test of 87.12 and a p-value of 6.15E-15, demonstrating a significant difference in performance among the algorithms. The average ranks indicate the relative efficiency of each algorithm, where lower ranks represent a better performance. Consequently, AOA, BAGNDO, and ChOA achieved the top average ranks, indication superior convergence behavior.

#### Ablation study

The ablation study asses the performance of the proposed BAGNDO after removing each components (ACRL, Elite, and GDWR). Table 10 show that the removing of the ARL strategy has the most significant performance degradation in most dataset, confirming that it play crucial role in increaing population diversity and preventing prematural convergence. The absence of Elite strategy also results in noticialbe degration in the average classification since it balance between exploration and exploitation and maintain the best individual across different iterations. Excluding GDWR results in moderate performance drop as it enhances the local explitation by guide the poor solution to the promising regions, overll, complete BAGNDO framework achieves consistent lowest classification error, demonstrating that the proposed component harmony work to improve robustness, convergence, and solution quality compared to basic GNDO and its ablated variants.

**Table 10:** Ablation study: average classification error.

DS. No	BAGNDO	–ACRL	–Elite	–GDWR	GNDO
1	**0.0067**	**0.0315**	**0.0248**	**0.0189**	0.0876
2	**0.1263**	**0.1652**	**0.1526**	**0.1408**	0.2144
3	**0.0123**	**0.0589**	**0.0417**	**0.0305**	0.1563
4	**0.2010**	**0.2564**	**0.2432**	**0.2251**	0.3120
5	0.2420	**0.2420**	**0.2420**	**0.2420**	0.2420
6	0.2852	**0.2926**	**0.2891**	**0.2874**	0.2859
7	0.2707	**0.2815**	**0.2768**	**0.2734**	**0.2672**
8	**0.2534**	**0.3786**	**0.3452**	**0.2987**	0.4551
9	**0.2649**	**0.2834**	**0.2746**	**0.2698**	0.2770
10	**0.2500**	**0.2918**	**0.2742**	**0.2627**	0.3190
11	**0.3400**	**0.4723**	**0.4386**	**0.3819**	0.5498
12	**0.4452**	**0.4589**	**0.4517**	**0.4483**	0.4503
13	**0.1960**	**0.2029**	**0.2003**	**0.1981**	0.2060
14	**0.2830**	**0.3158**	**0.3016**	**0.2919**	0.3466
15	**0.1040**	**0.1432**	**0.1289**	**0.1168**	0.1813
16	0.6530	**0.6562**	**0.6549**	**0.6537**	0.6546
17	**0.2449**	**0.2576**	**0.2518**	**0.2473**	0.2652
18	**0.0950**	**0.1984**	**0.1642**	**0.1216**	0.2871

Significant values are in [bold].

## Conclusion

This study successfully introduced a Binary Adaptive Generalized Normal Distribution Optimizer to address the critical challenge of feature selection in machine learning. The proposed algorithm strategically enhances the foundational GNDO by integrating an Adaptive Cauchy Reverse Learning strategy to prevent premature convergence, an Elite Pool to maintain a balanced search, and a Gaussian Distribution-based Worst-solution Repair method to refine poor solutions. The comprehensive empirical evaluation on 18 diverse datasets unequivocally validates the superiority of BAGNDO. Quantitatively, BAGNDO achieved the best mean fitness value across all datasets and secured the lowest classification error in 14 out of 18 cases. Furthermore, it consistently selected the smallest number of features in 14 datasets, effectively reducing dimensionality without compromising—and often enhancing—predictive accuracy. The non-parametric Wilcoxon test confirmed that BAGNDO’s performance was statistically significantly better than competing algorithms in most comparisons. The Friedman test ranked BAGNDO among the top performers with an average rank of 2. Despite its demonstrated excellence, a noted limitation is the algorithm’s computational demand, which may impact scalability on extremely large datasets. Future work will focus on address complex real-world problem such as intelligent early fault management^77^, few-shot defect detection^78^, smoothing and matrix decomposition^79^, and route planning under uncertainty^80^.

## Data Availability

All data generated or analyzed during this study are included in this published article.
